# Effects of Wearing Face Masks While Using Different Speaking Styles in Noise on Speech Intelligibility During the COVID-19 Pandemic

**DOI:** 10.3389/fpsyg.2021.682677

**Published:** 2021-06-28

**Authors:** Hoyoung Yi, Ashly Pingsterhaus, Woonyoung Song

**Affiliations:** ^1^Department of Speech, Language, and Hearing Sciences, Texas Tech University Health Sciences Center, Lubbock, TX, United States; ^2^Department of Educational Psychology and Leadership, Texas Tech University, Lubbock, TX, United States

**Keywords:** COVID-19, face masks, speech intelligibility, clear speech, audiovisual perception

## Abstract

The coronavirus pandemic has resulted in the recommended/required use of face masks in public. The use of a face mask compromises communication, especially in the presence of competing noise. It is crucial to measure the potential effects of wearing face masks on speech intelligibility in noisy environments where excessive background noise can create communication challenges. The effects of wearing transparent face masks and using clear speech to facilitate better verbal communication were evaluated in this study. We evaluated listener word identification scores in the following four conditions: (1) type of mask condition (i.e., no mask, transparent mask, and disposable face mask), (2) presentation mode (i.e., auditory only and audiovisual), (3) speaking style (i.e., conversational speech and clear speech), and (4) with two types of background noise (i.e., speech shaped noise and four-talker babble at −5 signal-to-noise ratio). Results indicate that in the presence of noise, listeners performed less well when the speaker wore a disposable face mask or a transparent mask compared to wearing no mask. Listeners correctly identified more words in the audiovisual presentation when listening to clear speech. Results indicate the combination of face masks and the presence of background noise negatively impact speech intelligibility for listeners. Transparent masks facilitate the ability to understand target sentences by providing visual information. Use of clear speech was shown to alleviate challenging communication situations including compensating for a lack of visual cues and reduced acoustic signals.

## Introduction

Coronavirus disease 2019 (COVID-19) dominated 2020 and the pandemic caused an unprecedented shut-down, resulting in the occurrence of drastic change in our daily lives and communities all over the world. COVID-19 is a transmissible disease caused by severe acute respiratory syndrome coronavirus 2 (SARS-CoV-2) and is highly contagious from person to person. As of May 4, 2021, confirmed infection cases exceeded 151 million, and the weekly epidemiological update, reported by the World Health Organization (WHO), stated that over 3 million deaths world-wide have been lost due to the COVID-19 pandemic (World Health Organization, 2021, May 4). COVID-19 affects/attacks the respiratory system and is primarily transmitted between people via the inhalation or ingestion of infectious respiratory droplets (>5–10 μm in diameter). Some research findings also indicate possible transmission through aerosol (<5 μm in diameter) (World Health Organization, 2020, July 9). Experimental and epidemiological data support the use of face masks to reduce the spread of SARS-CoV-2 (Cheng et al., 2020; Liang et al., 2020; Howard et al., 2021; Rader et al., 2021). Wearing face masks also reduces the emission of respiratory droplets containing other respiratory viruses such as seasonal human coronaviruses, influenza viruses, and rhino viruses (Leung et al., 2020). Covering both mouth and nose has a significant effect on filtering small particles across various types of face masks compared to not wearing face masks (Fischer et al., 2020; Konda et al., 2020; Clapp et al., 2021). The United States' Center for Disease Control and Prevention (CDC) recommends individuals wear face masks/coverings and abide by social distancing (> 2 feet) guidelines in public settings such as doctor's offices, schools, pharmacies, and other areas of significant community-based transmission (Centers for Disease Control Prevention, 2021). It is crucial that people wear face masks to reduce the spread of the virus in daily communication situations during the COVID-19 pandemic.

Unfortunately, studies have shown that face masks dampen speech acoustic signals and degrade the effect of verbal communication, which are two critical aspects of message intelligibility (Palmiero et al., 2016; Atcherson et al., 2017; Corey et al., 2020; Goldin et al., 2020; Magee et al., 2020). Face masks can result in detrimental effects on verbal communication and speech intelligibility by occluding important visual cues from mouth and lip gestures, interfering with natural articulatory movements, and altering speech acoustic features (Bond et al., 1989; Palmiero et al., 2016; Goldin et al., 2020). While speech communication is generally thought to be received only auditorily, the visual aspects of speech play a critical role in speech perception too. A prominent example of the interaction between audio and visual information to perceive speech is “McGurk effect” (McGurk and MacDonald, 1976). Auditory speech perception can be altered when an auditory speech utterance is incongruent with visual articulation (Tiippana, 2014). Visual speech cues such as mouth, lip, and tongue movements can provide temporal information about speech production (Chandrasekaran et al., 2009). The classical research studies of audiovisual speech demonstrated that visual speech cues complement auditory information to enhance speech intelligibility especially when target speech is interrupted by background noise (Sumby and Pollack, 1954; Erber, 1975). Recently, neural mechanisms for visual speech have been extensively examined in human and non-human primates. These studies have provided evidence that the auditory pathway plays a major role in visual speech processing by showing that silent lipreading activates the auditory cortical regions (see a review Campbell, 2008). Wearing face masks may negatively impact the quality of communication in face-to-face interactions due to the omission of visual speech cues such as lip movements. Currently, there are only a few studies outlining how the use of face masks affect communication by decreasing speech intelligibility in noisy environments and how talkers (i.e., speakers wearing face masks) could enhance their speech to improve intelligibility in this challenging communication environment (Keerstock et al., 2020; Magee et al., 2020). Thus, it is imperative to examine the effects of wearing face masks on communication.

Although current literature is limited regarding the effects of face masks on speech intelligibility in the presence of noise, newer research has revealed relevant findings concerning how facial coverings attenuate acoustic signals and decrease speech intelligibility. Corey et al. (2020) evaluated how different mask types affect acoustic signals. They found that acoustic signals above 4 kHz were attenuated the most regardless of the type of mask worn by the talker. Disposable surgical face masks offered the best acoustic performance, whereas transparent masks and shields presented the greatest acoustic attenuation. Magee et al. (2020) evaluated how face masks affect acoustic signals and speech perception at the single-word and sentence levels. Consistent with Corey et al. (2020), Magee and colleagues found that different types of face masks affected acoustic signals differently, with higher frequencies of acoustic signals being attenuated across three types of masks (i.e., surgical, cloth, N95). Additionally, the listeners identified target words produced by a talker, in a quiet listening environment where intelligibility remained above ninety-two percent regardless of the face mask worn by the talker. A study presented at a conference by Keerstock et al. (2020) evaluated how protective masks, background noise, and non-native accents affect speech intelligibility and overall communication. They found speech produced in conversational speaking styles with a disposable face mask was as intelligible as speech produced without a mask. More specifically, conversational speech produced while wearing a disposable face mask did not negatively affect speech intelligibility in quiet listening conditions for either native or non-native speakers and occasionally even in the presence of noise for the native speaker. However, foreign accented sentences produced by a non-native speaker wearing a mask reduced speech intelligibility compared to sentences produced by the same speaker who wore no mask in the presence of background noise.

**Figure 1:**
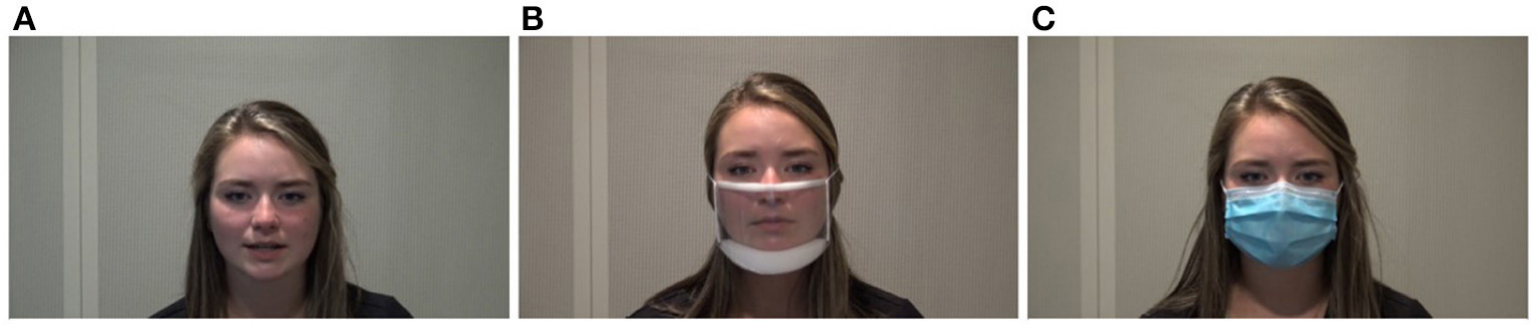
Face mask conditions including **(A)** no mask, **(B)** transparent mask, and **(C)** disposable face mask.

The current study evaluated the impact of two types of face masks including a non-medical disposable face mask (referred to as “disposable face mask”) and a transparent mask (i.e., ClearMask) on communication. Figure 1 presents the two types of face masks used in this study. The non-medical disposable face mask is made of three layers of filtration that includes non-woven fabric, melt blown, and an additional filter. To protect people from exposure to infectious droplets, the CDC advised individuals to wear non-medical disposable face masks as their Personal Protective Equipment (PPE) which is comparable to disposable medical face masks (i.e., surgical mask) in April of 2020 (Centers for Disease Control Prevention, 2020). At the beginning of the COVID-19 pandemic, all types of face masks were not easily accessible to people and the CDC recommended not using surgical masks to save the medical supplies for the medical setting. Non-medical disposable face masks are similar to surgical masks regarding the materials of the three layers of filtration, and they are widely available in public. Previous research found that disposable medical face masks attenuate the lowest amount of acoustic signals, ~3–4 decibels (dB) at higher frequencies, compared to a variety of other face masks (Corey et al., 2020; Goldin et al., 2020). Although we did not use disposable *medical* face masks, their findings are applicable to disposable non-medical face masks due to the similarities between medical and non-medical disposable face masks.

The transparent mask (i.e., ClearMask) has a transparent, plastic barrier that enables listeners to observe a speaker's facial expressions and mouth and lip movements during speech production. We selected the transparent mask because the mask has an anti-fog feature that is different to the other available transparent masks. Additionally, this mask offers more visibility of the talker's face and it meets American Society for Testing and Materials (ASTM) level 3 requirements that indicates a high level of barrier protection (ASTM F3502-21, 2021). In addition to disposable face masks, the CDC also recommends using transparent masks for specific populations. Transparent masks should be worn when interacting with individuals who are deaf or hard of hearing, young children or students learning to read, students learning a new language, individuals with disabilities, and/or individuals who need to see the proper shape of the mouth for understanding specific consonant and vowel sounds.

A recent study (Kratzke et al., 2021) evaluated the effect of cover masks vs. transparent masks on patient-surgeon relationships. Researchers evaluated how patients perceive surgeons who wore transparent masks. The patients showed a preference for seeing their surgeons' faces through a transparent mask as opposed to a surgical mask. Patients rated their surgeons who wore transparent masks as being better communicators and having more empathy. Although transparent masks attenuate acoustic signals greater than other face masks (Corey et al., 2020; Goldin et al., 2020), visual information from the speaker's facial expressions and lip movements improves speech intelligibility (Atcherson et al., 2017). Providing visual information or preventing the occlusion of visual cues can be an effective solution for individuals with communication disorders or for individuals who heavily rely on visual information to interpret messages (Erber, 1975; Kaplan et al., 1987; Schwartz et al., 2004; Tye-Murray et al., 2007; Jordan and Thomas, 2011; Atcherson et al., 2017). Atcherson et al. (2017) found that listeners with and without communication disorders (i.e., hearing-loss) benefitted from the visual input offered by a transparent mask based on the comparison between auditory only (AO) and audiovisual (AV) presentations. The use of transparent masks may be beneficial for verbal communicative exchanges during the COVID-19 pandemic where social distancing and face masks are highly recommended or required.

Aside from wearing transparent masks, the use of clear speech could be an additional solution to combat the effects of wearing face masks and could further improve speech intelligibility. Clear speech, known as listener-oriented clear speech, is a type of speaking style adaptation in which talkers adjust their output in response to communication challenges, such as talking to a listener who has difficulty understanding (i.e., individuals with hearing impairments or second language learners) (Smiljanić and Bradlow, 2009; Calandruccio et al., 2010). It has been well-established that clear speech enhances speech intelligibility by increasing acoustic-articulatory outcomes (Smiljanić and Bradlow, 2009; Cooke et al., 2014; Smiljanic and Gilbert, 2017a,b). Clear speech has been shown to improve speech intelligibility in the various populations: second language learners (Bradlow and Bent, 2002), learning-impaired children (Bradlow et al., 2003), individuals with hearing loss (Ferguson, 2012; Ferguson and Quené, 2014), children with cochlear implants (CIs) (Smiljanic and Sladen, 2013), and adult with CIs (Rodman et al., 2020) even though the intelligibility enhancement of clear speech varies depending on the listeners groups (Ferguson and Quené, 2014; Smiljanic and Gilbert, 2017a,b) and contexts (Van Engen et al., 2014). With accumulated evidence of clear speech enhancement for speech intelligibility, we expect clear speech benefits to improve overall communication in the COVID-19 pandemic.

In addition to the communication barrier presented by face masks, the presence of background noise degrades perceptual access in verbal communicative exchanges by interfering with listening conditions and reducing signal to noise ratios (Bond et al., 1989; Palmiero et al., 2016). Face-to-face communication occurs in environments with differing noise levels. These noise levels fall on a natural quiet-noisy continuum. When we encounter noisy environments, different background noises can interfere with speech signals (i.e., energetic masker, informational masker). Listeners rely extensively on seeing talkers' articulatory movements and facial expressions to understand spoken words in noisy environments (Sumby and Pollack, 1954; Erber, 1975; Buchan et al., 2008). The combination of background noise and required facial coverings can increase the difficulty for people to communicate effectively and efficiently. Because of this common interference of noise on verbal communication, the present study investigated the effects of using clear speech with a transparent mask in different types of noises, including Speech-Shaped Noise (SSN) and 4-talker babble (4-T). SSN is classified as an energetic masker (EM) that occurs in the auditory periphery and interferes with the perception of speech stimuli due to spectro-temporal overlap of the target signal and the masker (i.e., white noise). Four-talker babble is considered as an informational masker (IM) that occurs due to lexical interference and associated cognitive load (Cooke et al., 2008). Benefits of clear speech have also been investigated in different presentation modalities across various background noises (Van Engen et al., 2014). Van Engen et al. (2014) further revealed that clear speech provided greater benefits for listeners given AV input compared to AO input in the presence of 4-T and 8-talker babble (8-T). Based on the findings in Van Engen et al. (2014), we seek to further examine whether a clear speech style could be a potential tool for individuals to utilize in the presence of difficult maskers (i.e., noisy environments) and COVID-19 regulations (i.e., facial coverings and social distancing).

The goal of this project was to investigate the effects of wearing face masks on speech intelligibility to listeners in the presence of background noise. Furthermore, we examined the benefits of using clear speech and wearing a transparent face mask on speech intelligibility. This examination can enhance our understanding of factors influencing audiovisual speech perception in this challenging environment. We aim to provide health-care professionals with valuable information and evidence for communication recommendations to improve speech intelligibility during and after the COVID-19 pandemic.

## Materials and Methods

### Participants

Twenty-six adults (Female 24, male 2) between the ages of 18 and 47 (median age: 21) were recruited from the Texas Tech University Health Sciences Center (TTUHSC) and Lubbock, Texas geographic area. All participants were TTUHSC students except one. All participants were native speakers of American English. Two participants reported their home language was Spanish. While their home language might affect experimental results, their performances were found to be comparable with the other participants. All participants passed a hearing-screening test for hearing sensitivity of <25 dB hearing level at 500, 1,000, 2,000, and 4,000 Hz in both ears. All participants gave written informed consent and student-participants received extra credits in a course as compensation for their time. All experimental procedures followed the TTUHSC COVID-19 protocol and were approved by the Institutional Review Board at TTUHSC.

### Materials

#### Sentences and Recordings

Target sentences were video recorded by the second author of the current study, a female, native speaker of American English. The set of stimuli consisted of 120 sentences based on sentences from the Basic English Lexicon (BEL) (Calandruccio and Smiljanic, 2012). The list of target sentences is provided in Supplementary Material. Each sentence contained four keywords (e.g., “The sick person feels better”). All sentences were produced by the talker wearing no face mask and subsequently when wearing two different face masks (i.e., transparent mask, disposable face mask). Figure 1 displays the no face mask condition and the two different types of face masks used in this study. The speaker produced sentences using clear and conversational speaking styles. She produced conversational speech first followed by clear speech. For conversational speech, the talker was instructed to speak as if she was talking to someone familiar. For clear speech, the speaker was asked to talk as if she was speaking to someone who has trouble understanding her due to a hearing impairment.

In effort to prevent a *practice effect*, an alteration in task performance due to increased practice and familiarity, when recording the same target sentences across different mask conditions, the tasks were organized and completed in a particular order. Specifically, 120 target sentences were equally divided into three sets and each set was produced with a different rotation of mask condition (i.e., set A: no mask > disposable face mask > transparent mask, set B: disposable face mask > transparent mask > no mask, and set C: transparent mask > no mask > disposable face mask). Each rotation per set was recorded using conversational speech and clear speech. Sentences were video recorded using a Sony FDR-AX33 camera. Audio recordings were made at a sampling rate of 44.1 k Hz using a DPA 4060 Mini omnidirectional microphone, which was placed on a table stand in front of the talker. An audio interface, Focusrite Scarlett 2i2 (2nd Gen), was used to enhance the quality of the recordings.

Audio recordings were accurately segmented into individual sentences by the third author using the TextGrid function in Praat (Boersma and Weenink, 2021) which was used for annotation, labeling each segmented sentence on the sound files. A total of 720 sentences (120 sentences ×3 types of face masks ×2 speaking styles) were equated to 60 dB sound pressure level (SPL) by average root-mean-squared amplitude. Two types of maskers were mixed with target sentences. Speech-Shaped Noise (SSN) was generated by filtering white noise to the long-term average spectrum from a set of 80 sentences (Gilbert et al., 2014). Four-talker babble (4-T) tracks were created by four female speakers of American English. They produced a set of 60 meaningful English sentences (Bradlow and Alexander, 2007) in a sound-attenuated booth. Audio recordings for the multi-talker babbles and target sentences in this study were completed using the same equipment. Target sentences were digitally mixed with maskers at a SNR of −5 dB SPL. The SNR level was determined through piloting to avoid ceiling effect. Each of the stimulus files consisted of 500 ms of noise, followed by the speech-plus-noise files, and ending with 500 ms of only noise. The noise preceding and following the speech stimuli was equivalent to the level of the noise mixed with the speech. All manipulation of audio stimuli was performed by Praat. After the video recordings were synchronized with the audio recordings using Openshot 2.5.1, each audiovisual file was cut and rendered with each audio stimulus to 1,920 × 1,080 resolution and 30 FPS video with 44.1 k Hz 32bit PCM audio using MoviePy 1.0.3 in Python.

#### Acoustic Features of the Sentence Recordings

A series of acoustic analyses were performed on all sentences to assess how the talker's speech was acoustically different in two speaking styles across the three types of face mask conditions. F0 mean (Hz), F0 range (Hz), and sentence duration (millisecond, ms) were measured to identify acoustic characteristics across the different conditions of speaking styles and face masks. The results of these three acoustic measurements are given in Table 1. All acoustic features appeared with no differences across the three face mask types. CL sentences led to longer sentence duration and greater F0 ranges compared to CO sentences. F0 mean showed no differences which might be due to the lower minimum F0 and the higher maximum F0 for CL sentences compared to CO sentences. According to the analysis of the acoustic features, the speaker produced clear sentences with much slower and exaggerated pitch ranges.

**Table 1:** Acoustic measures of sentence materials as produced with no mask (NO), transparent mask (TM), and disposable face mask (DM), in conversational speech and in clear speech.

**Speech style**	**Face mask**	**f0mean (Hz)**	**f0range (Hz)**	**Duration (ms)**
Conversational	NO	225.03	113.35	1638.24
Conversational	TM	223.84	115.58	1609.41
Conversational	DM	224.14	118.09	1584.13
Clear	NO	221.69	132.92	3069.72
Clear	TM	222.55	140.20	3218.86
Clear	DM	221.53	138.87	3174.53

**Figure 2:**
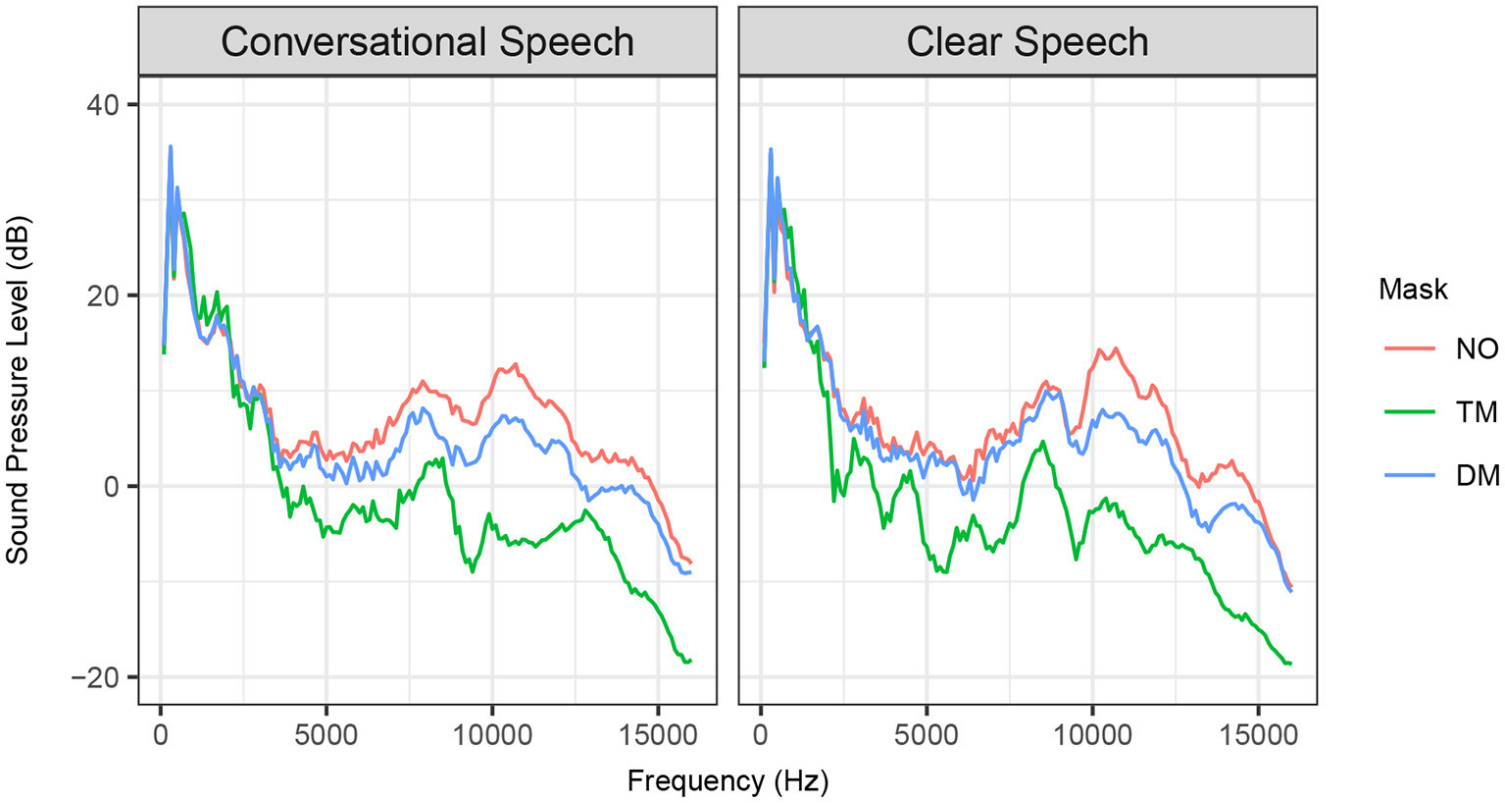
Long-term-average spectrum (LTAS) for conversational speech (left) and clear speech (right) produced with no mask (NO, red line), transparent mask (TM, green line), and disposable face mask (DM, blue line).

Long-term-average spectrum (LTAS) analysis was completed for each speaking style and mask types at a bandwidth of 100 Hz. The speaker's spectra varied across each face mask with large differences noted at the higher frequencies (see Figure 2). The transparent mask and disposable face mask used in our study attenuated frequencies above 3 kHz, which closely compares to the findings in previous research (Corey et al., 2020; Goldin et al., 2020; Magee et al., 2020). As expected, the transparent mask degraded more acoustics than the disposable face mask. We found that clear speech compensated for the attenuated acoustic signals in the disposable face mask condition, unlike conversational speech. Clear speech preserved the sounds in the 3–10 kHz frequency range by showing reduced variations between the no mask and disposable face mask conditions. The transparent mask did not show any conceivable changes from conversational speech to clear speech because both speaking styles showed large, comparable attenuation levels.

### Procedure

After passing the hearing screening and providing informed consent for the study, participants sat in front of a computer monitor. The distance between a computer monitor and participant was ~50 cm (20 inches), but the distance varied depending on participants' comfortability. Audio stimuli were played over Sennheiser HD 300 PRO Closed-back Professional Monitor Headphones at a comfortable listening level set by the experimenter. Visual stimuli were presented on a 14-inch Dell laptop. Instructions and stimuli were presented using E-PRIME 3.0 (Schneider et al., 2016). Participants were instructed to listen to each sentence and enter what they heard on the computer. Each trial was presented only once, but participants were offered an unlimited amount of time to type sentences and they controlled when to proceed to the next stimulus.

Participants completed a total of 132 trials including 12 practice items. A 120 target sentences were randomly selected out of a total pool of 720 sentences without repetition for each participant. Participants completed 24 different combinations of the four conditions. Thus, five sentences with 20 keywords were presented per each combination of presentation mode (AO, AV), speech style (CO, CL), type of face mask (no mask, transparent, disposable), and noise type (SSN, 4-T Babble). After practice trials, selected 120 sentences were randomly presented in two blocks (60 sentences per block) and participants were allowed to take a break as long as they wanted between the two blocks to minimize the effects of fatigue. Responses were scored by keywords correctly identified out of 480 words. Keywords with added or omitted morphemes were scored as incorrect.

## Results

Prior to statistical analysis, the proportions of keyword identification were descriptively analyzed. Overall, listeners performed better in the no mask condition (mean: 62.86%, standard deviation (SD): 26.66%) compared to the disposable face mask (mean: 41.92%, SD: 24.26%), and transparent mask (mean: 45.12%, SD: 30.58%) conditions. They also correctly identified more words in the AV mode (mean: 62.90%, SD: 26.18%) compared to the AO mode (mean: 37.04%, SD: 25.21%). Listeners performed better for clear speech (mean: 60.08%, SD: 29.01%) over conversational speech (mean: 39.86%, SD: 24.70%) and SSN (mean: 63.22%, SD: 22.75%) over 4-T (mean: 36.71%, SD: 28.05%).

Word identification data were analyzed with a mixed effects logistic regression model using the lme4 package (v1.1-14: Bates et al., 2015) in R (v3.6.3). Keyword identification (i.e., correct or incorrect) was the dichotomous dependent variable. Fixed effects included type of face masks (no mask, transparent, disposable face mask), presentation mode (AO, AV), speech style (CO, CL), noise type (SSN, 4-T babble), and interactions of all possible combinations among the main factors. To account for baseline differences in word identification across participants and sentences, we included by-participant and by-sentence intercepts as random effects. The reference levels were the no mask, CO, AO, and SSN. Tables 2, 3 demonstrate the result of the mixed effects logistic regression model. Table 2 shows parameter estimates of odds ratio for the main effects and interaction effects. Table 3 includes the random effects. Type III Wald chi-square tests were used to examine the overall effect of fixed factors in the mixed effects logistic regression model. The results from this analysis revealed significant main effects of type of face mask [χ^2^ (2) = 67.550, *p* < 0.001], presentation mode, [χ^2^ (1) = 117.098, *p* < 0.001], speech style [χ^2^ (1) = 9.897, *p* = 0.002], and noise type [χ^2^ (1) = 50.991, *p* < 0.001]. The significant main effects denote listeners performed better for clear sentences produced with no mask presented in the AV mode in SSN. There were significant two-way interactions between type of face mask and presentation mode [χ^2^ (2) = 15.784, *p* < 0.001], type of face mask and speaking style [χ^2^ (2) = 18.619, *p* < 0.001], type of face mask and noise type [χ^2^ (2) = 9.969, *p* = 0.007], presentation mode and speaking style [χ^2^ (1) = 19.664, *p* < 0.001], and speaking style and noise type [χ^2^ (1) = 11.078, *p* = 0.001]. The model also revealed significant three-way interactions among type of face mask, presentation mode, and speaking style [χ^2^ (2) = 29.078, *p* < 0.001], type of face mask, speaking style, and noise type [χ^2^ (2) = 10.586, *p* = 0.005], and presentation mode, speaking style and noise type [χ^2^ (1) = 6.904, *p* = 0.009]. All other two-way and three-way interactions were not significant.

**Table 2:** Parameter estimates of odds ratio for the main effects and interaction effects of the mixed effects logistic regression model.

**Effects**	**Estimate**	**Std. error**	**Odd Ratio**	***Z* value**	***P*-value**
Intercept	−0.999	0.127	0.368	−7.863	<0.001
**Speaking style (Ref. conversational)**					
Clear	0.441	0.140	1.555	3.146	0.002
**Face mask (Ref. no)**					
Transparent	−1.431	0.186	0.239	−7.688	<0.001
Disposable	−0.892	0.166	0.410	−5.365	<0.001
**Noise type (Ref. 4-talker babble)**					
Speech-shaped	0.986	0.138	2.680	7.141	<0.001
**Presentation mode (Ref. audio only)**					
Audiovisual	1.523	0.141	4.585	10.821	<0.001
Speech style * face mask (Ref. conversational and no)					
Clear * transparent	−0.705	0.272	0.494	−2.591	0.010
Clear * disposable	0.518	0.217	1.678	2.389	0.017
Speech style * noise type (Ref. conversational and 4-talker babble)					
Clear * speech-shaped	0.659	0.198	1.932	3.328	0.001
Face mask * noise type (Ref. no and 4-talker babble)					
Transparent * speech-shaped	0.676	0.230	1.966	2.943	0.003
Disposable * speech-shaped	0.468	0.212	1.597	2.206	0.027
Speech style * presentation mode (Ref. conversational and audio only)					
Clear * Audiovisual	0.945	0.213	2.573	4.434	<0.001
Mask type * presentation mode (Ref. no and audio only)					
Transparent * audiovisual	0.132	0.232	1.141	0.570	0.569
Disposable * audiovisual	−0.739	0.218	0.478	−3.394	0.001
Noise type * presentation mode (Ref. 4-talker babble and audio only)					
Speech-shaped * Audiovisual	−0.258	0.200	0.773	−1.289	0.198
Speech style * mask type * noise type (Ref. conversational, no, and 4-talker babble)					
Clear * transparent * speech-shaped	0.734	0.335	2.084	2.195	0.028
Clear * disposable * speech-shaped	−0.386	0.292	0.680	−1.324	0.186
Speech Style * mask type * presentation mode (Ref. conversational, no, and audio only)					
Clear * transparent * audiovisual	0.699	0.345	2.011	2.027	0.043
Clear * disposable * audiovisual	−1.102	0.304	0.332	−3.622	<0.001
Speech style * noise type * presentation mode (Ref. conversational, 4-talker babble, and audio only)					
Clear * speech-shaped * audiovisual	−0.837	0.319	0.433	−2.628	0.009
Mask type * noise type * presentation mode (Ref. no, 4-talker babble, and audio only)					
Transparent * speech-shaped * audiovisual	−0.012	0.305	0.988	−0.040	0.968
Disposable * speech-shaped * audiovisual	−0.459	0.293	0.632	−1.567	0.117
Speech * mask * noise * presentation (Ref. conversational, no, 4-talker babble, and audio only)					
Clear * transparent * speech-shaped * audiovisual	0.117	0.481	1.124	0.242	0.809
Clear * disposable * speech-shaped * audiovisual	1.146	0.432	3.146	2.655	0.008

**Table 3:** Random effects of the mixed effects logistic regression model.

**Random effects**	**Variance**	**Observations**
Intercept | subjects	0.090	*n* = 26
Intercept | sentence number	0.257	*n* = 120

**Figure 3:**
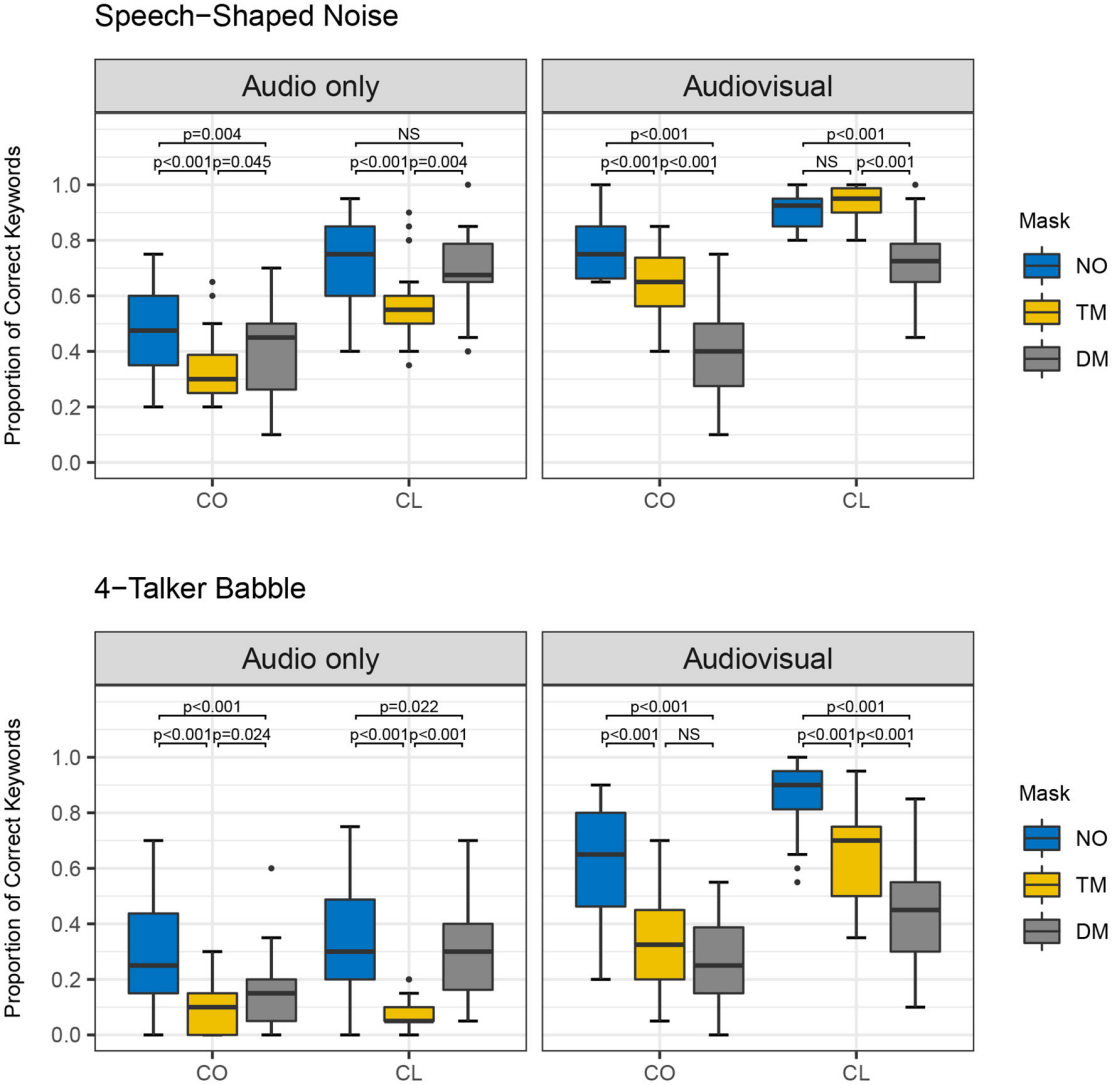
Proportion of correct keywords in sentences for comparisons of no mask (NO), transparent mask (TM), and disposable face mask (DM) produced with conversational (CO) and clear speaking (CL) styles presented in Audio only and Audiovisual modes with Speech-Shaped Noise and 4-Talker Babble.

**Figure 4:**
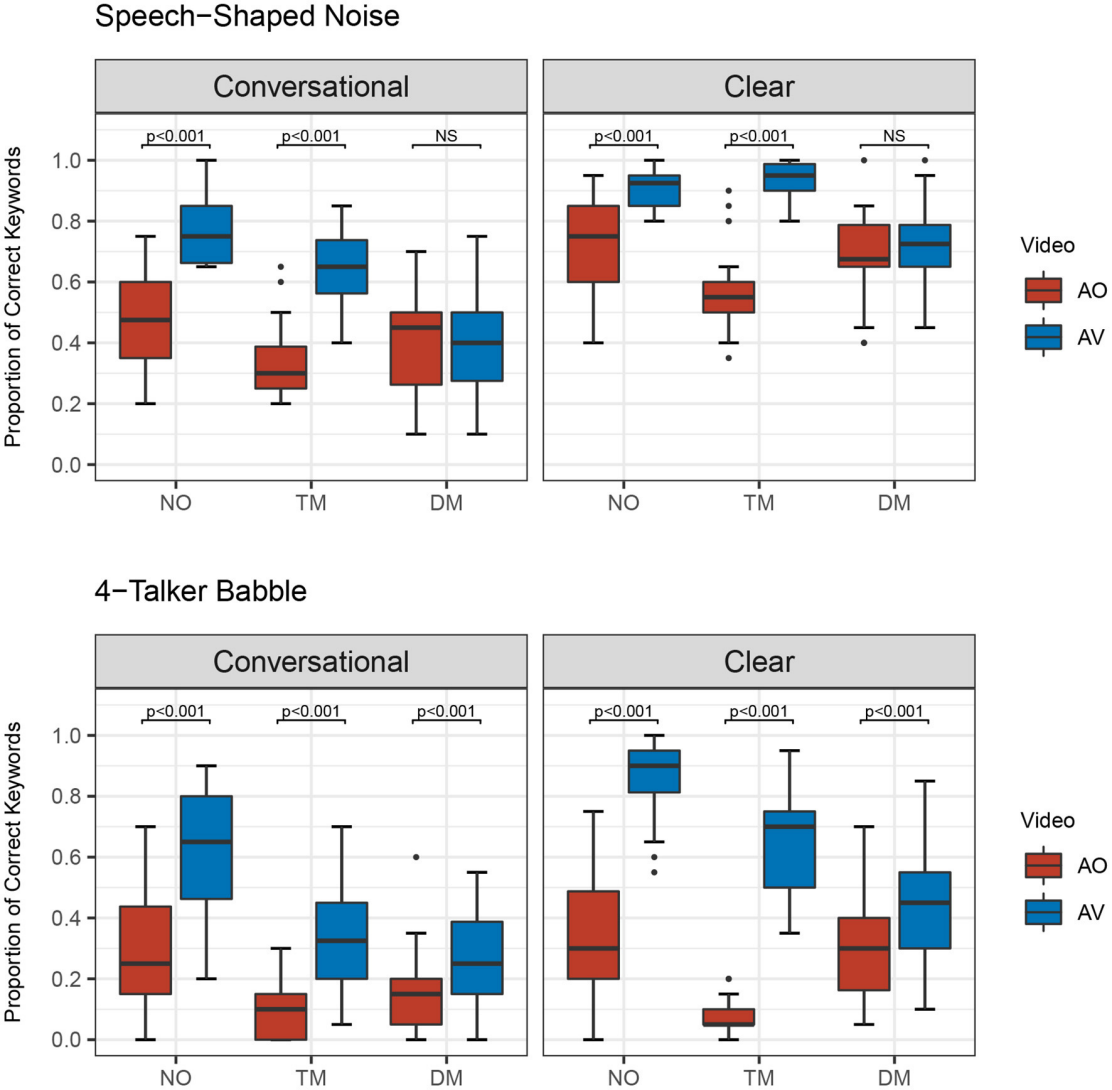
Proportion of correct keywords in sentences for comparisons of Audio only (AO) and Audiovisual (AV) modes for each type of face mask (no mask: NO, transparent mask: TM, disposable face mask: DM) in both background noises and speaking styles.

**Figure 5:**
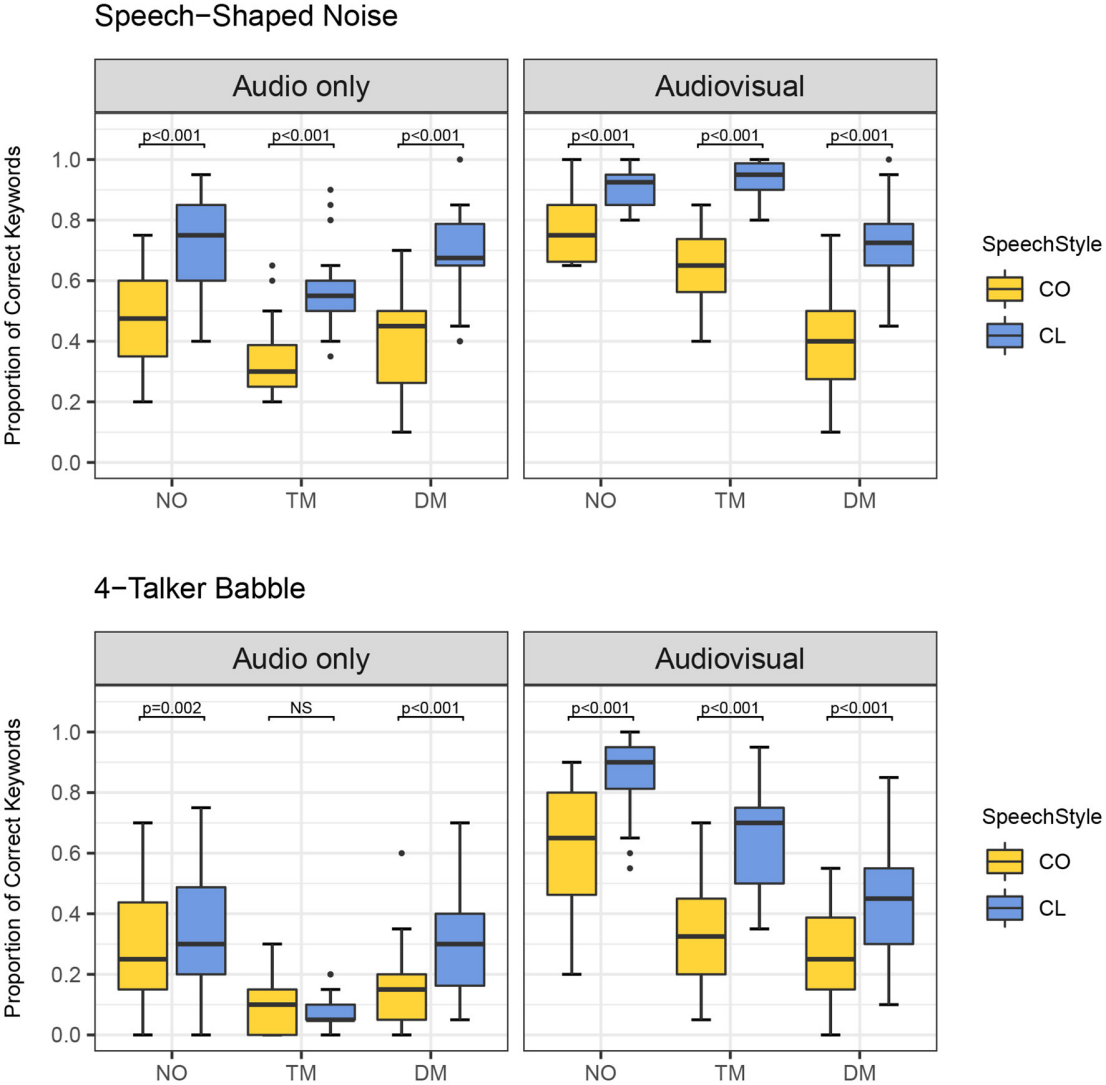
Proportion of correct keywords in sentences for comparisons between conversational (CO) and clear (CL) in each type of face masks (no mask: NO, transparent mask: TM, disposable face mask: DM) in both background noises and presentation modes.

Furthermore, there was a significant four-way interaction among the main factors [χ^2^ (2) = 8.517, *p* = 0.014]. This finding indicates all the lower-level significant interactions differed by the other factors. For example, the significant two-way interaction of type of face mask and presentation mode varied depending upon speaking style and noise type. Figures 3–5 show the proportion of keyword identification for each level in the four conditions. To further analyze the significant four-way interactions, the comparisons of speaking style, face mask, presentation mode, and background noise were tested within each level of the other factors by Bonferroni's correction using the “emmeans” function. The results of *post-hoc* analyses are reported in Tables 4–7.

**Table 4:** The contrast of face masks (no mask, NO, transparent mask, TM, and disposable face mask, DM) in each speaking style, background noise, and presentation mode.

**Presentation**	**Noise**	**Speech style**	**Contrast mask**	**Estimate**	**SE**	***Z***	***P*-value**
Audio only	4-T babble	Conversational	NO – TM	1.431	0.186	7.688	<0.001
			NO – DM	0.892	0.166	5.365	<0.001
			TM - DM	−0.539	0.203	−2.659	0.024
		Clear	NO – TM	2.136	0.199	10.740	<0.001
			NO – DM	0.374	0.140	2.677	0.022
			TM - DM	−1.762	0.202	−8.735	<0.001
	SSN	Conversational	NO – TM	0.755	0.135	5.591	<0.001
			NO – DM	0.424	0.132	3.205	0.0041
			TM - DM	−0.331	0.136	−2.434	0.045
		Clear	NO – TM	0.726	0.140	5.166	<0.001
			NO – DM	0.292	0.144	2.033	0.126
			TM - DM	−0.433	0.136	−3.176	0.005
Audiovisual	4-T babble	Conversational	NO – TM	1.299	0.138	9.413	<0.001
			NO – DM	1.631	0.142	11.511	<0.001
			TM - DM	0.332	0.144	2.314	0.062
		Clear	NO – TM	1.305	0.161	8.084	<0.001
			NO – DM	2.215	0.160	13.816	<0.001
			TM - DM	0.910	0.135	6.760	<0.001
	SSN	Conversational	NO – TM	0.635	0.146	4.354	<0.001
			NO – DM	1.622	0.144	11.231	<0.001
			TM - DM	0.987	0.135	7.288	<0.001
		Clear	NO – TM	−0.209	0.233	−0.900	1
			NO – DM	1.446	0.188	7.674	<0.001
			TM - DM	1.656	0.199	8.319	<0.001

*Summary of Post-hoc analysis for the significant four-way interaction with Bonferroni's correct*.

**Table 5:** The contrast of presentation modes (audio-only, AO and audiovisual, AV) in each face mask condition, background noise, and speaking style.

**Noise**	**Speech style**	**Mask**	**AO - AV**	**SE**	***Z***	**P-value**
4-T babble	Conversational	NO	−1.523	0.141	−10.821	<0.001
		TM	−1.655	0.184	−9.007	<0.001
		DM	−0.784	0.166	−4.711	<0.001
	Clear	NO	−2.468	0.161	−15.317	<0.001
		TM	−3.299	0.199	−16.563	<0.001
		DM	−0.627	0.138	−4.529	<0.001
SSN	Conversational	NO	−1.265	0.143	−8.867	<0.001
		TM	−1.385	0.138	−10.023	<0.001
		DM	−0.067	0.134	−0.495	0.621
	Clear	NO	−1.373	0.190	−7.245	<0.001
		TM	−2.308	0.195	−11.819	<0.001
		DM	−0.219	0.142	−1.546	0.122

*Summary of Post-hoc analysis for the significant four-way interaction with Bonferroni's correction*.

**Table 6:** The contrast of speaking styles (conversational, CO, and clear, CL) in each face mask condition, background noise, and presentation mode.

**Presentation**	**Noise**	**Mask**	**CO - CL**	**SE**	***Z***	***P*-value**
Audio only	4-T babble	NO	−0.441	0.140	−3.146	0.002
		TM	0.264	0.233	1.132	0.258
		DM	−0.959	0.165	−5.807	<0.001
	SSN	NO	−1.100	0.139	−7.887	<0.001
		TM	−1.129	0.136	−8.285	<0.001
		DM	−1.232	0.137	−8.986	<0.001
Audiovisual	4-T babble	NO	−1.386	0.161	−8.586	<0.001
		TM	−1.380	0.138	−9.968	<0.001
		DM	−0.802	0.139	−5.756	<0.001
	SSN	NO	−1.208	0.192	−6.303	<0.001
		TM	−2.053	0.197	−10.427	<0.001
		DM	−1.384	0.140	−9.914	<0.001

*Summary of Post-hoc analysis for the significant four-way interaction with Bonferroni's correction*.

**Table 7:** The contrast of background noises (4-T babble and speech-shaped noise, SSN) in each face mask condition, speaking style, and presentation mode.

**Presentation**	**Speech style**	**Mask**	**4-T - SSN**	**SE**	***Z***	***P-*value**
Audio only	Conversational	NO	−0.986	0.138	−7.141	<0.001
		TM	−1.661	0.183	−9.062	<0.001
		DM	−1.454	0.161	−9.021	<0.001
	Clear	NO	−1.644	0.142	−11.541	<0.001
		TM	−3.054	0.198	−15.405	<0.001
		DM	−1.726	0.142	−12.189	<0.001
Audiovisual	Conversational	NO	−0.728	0.145	−5.015	<0.001
		TM	−1.391	0.138	−10.058	<0.001
		DM	−0.736	0.141	−5.233	<0.001
	Clear	NO	−0.550	0.204	−2.691	0.007
		TM	−2.064	0.196	−10.519	<0.001
		DM	−1.318	0.140	−9.450	<0.001

*Summary of Post-hoc analysis for the significant four-way interaction with Bonferroni's correction*.

First, the comparisons among the mask conditions in each speaking style, presentation mode, and noise type were tested to find where the significant four-way interactions occurred. In the AO condition, for both speaking styles and background noises, word identification scores decreased significantly from the no mask to the disposable face mask, and to the transparent mask conditions (all *p-*values < 0.05, see Figure 3 and Table 4). In AO, only one comparison between no mask and disposable face mask for clear speech in SSN was not significantly different [β = 0.292, *SE* = 0.144, *Z* = 2.033, *p* = 0.126]. In the AV condition, all comparisons of the face masks for both speaking styles and background noises, word identification scores decreased significantly from the no mask to the transparent mask, and to the disposable face mask conditions (all *p-*values < 0.001) except in two comparisons. In the AV condition, clear speech between the transparent mask and no mask condition presented in SSN did not show a significant difference in word identification [β = −0.209, *SE* = 0.233, *Z* = −0.900, *p* = 1.000]. In the AV condition, conversational speech between transparent masks and disposable face masks presented with 4-T did not show a significant difference in word identification [β = 0.332, *SE* = 0.144, *Z* =2.314, *p* = 0.062].

Second, *post-hoc* analysis of the comparisons between AO and AV for each type of face mask in each of the background noises and speaking styles were examined (see Figure 4 and Table 4). Listeners performed significantly higher in AV than in AO mode for both background noises and speaking styles (all *p-*values < 0.001) except in both clear and conversational sentences produced with a disposable face mask in SSN [CO: β = −0.0665, *SE* = 0.134, *Z* = −0.495, *p* = 0.6205; CL: β = −0.219, *SE* = 0.142, *Z* = −1.546, *p* = 0.122].

Third, in the comparisons between CO and CL (see Figure 5 and Table 6), both the presentation modes, and background noises, the effect of clear speech was significant for the three types of mask conditions (no mask with 4-T babble in AO, *p* = 0.002; all the other contrasts of clear speech effect, *p* < 0.001) except the transparent mask presented in 4-T babble in AO [β = 0.264, *SE* = 0.233, *Z* = 1.132, *p* = 0.258].

Lastly, we examined listeners' performances between SSN and 4-T in each mask condition, speaking style, and presentation mode (see Table 7). Keywords presented in SSN showed significantly higher accuracy compared to the keywords presented in 4-T for both speaking styles, presentation modes, and all three mask conditions (No mask, clear speech in AV: *p* = 0.007, all other comparisons: *p-*values < 0.001).

## Discussion

The current study examined three specific areas of interest: (1) The impact of face masks on speech intelligibility in the presence of noise; (2) The benefits of wearing transparent masks on speech intelligibility; (3) The benefits of using clear speech to improve speech intelligibility when a speaker wears different masks. Overall, we found adverse effects of using face masks on speech intelligibility in the presence of background noise. When presenting both visual and auditory information, speech intelligibility improved in both the no mask and transparent mask conditions with both speaking styles and masker presentations. Clear speech enhanced speech intelligibility over conversational speech in most cases.

### The Impact of Face Masks on Speech Intelligibility in the Presence of Noise

Our findings revealed the use of face masks (i.e., disposable face masks and transparent masks) decreases speech intelligibility compared to no mask in the presence of background noise. These results outline the consequences of background noise combined with the presence or absence of face masks on speech intelligibility. The findings regarding the adverse effects of using face masks on speech intelligibility in the current study was slightly different from the findings in previous research (Keerstock et al., 2020; Magee et al., 2020). Magee et al. (2020) measured speech intelligibility in a quiet listening environment without background noise. Keerstock et al. (2020) used 6T presented at SNR of +5 and 0 dB. The current study applied 4-T and SSN presented at −5 dB SNR. These different task procedures may contribute to discrepant results among the studies. Magee and colleagues' results indicated that speech intelligibility is not significantly impacted by the use of face masks in quiet listening environments. However, they did not evaluate how the presence of noise could impact speech intelligibility given the type of mask employed in their study (Magee et al., 2020). Keerstock et al. (2020) found no significant differences in speech intelligibility when a native talker used conversational speech with and without a disposable face mask across diverse listening conditions. However, Keerstock et al. (2020) also revealed negative effects of a disposable face mask on intelligibility when native speakers listened to foreign-accented sentences presented in background noise. These diverse research designs indicate that wearing face masks reduces speech intelligibility in challenging communicative environments including in background noise and where foreign accented speech is presented to a listener.

The current study increased the difficulty of listening conditions compared to those in Keerstock et al. (2020). We found a significant difference in speech intelligibility between the two types of background noise (i.e., energetic masker and informational masker) regardless of the presence of a face mask or the type of mask worn. The pure energetic masker (i.e., SSN) proved to be less challenging for listeners to understand the talker compared to informational maskers (i.e., multi-talker babble) for both the transparent mask and disposable face mask conditions. Informational maskers induce greater attentional and higher-order cognitive cost to selectively pay attention to the target sentences while inhibiting the competing linguistic information from maskers (Simpson and Cooke, 2005; Cooke et al., 2008; Schoof and Rosen, 2014; Van Engen et al., 2014). Thus, informational masking, often experienced in community listening environments, may increase the chances of miscommunication and poorer speech intelligibility while they are wearing a face mask. Our findings illuminate the presence of a problem in our current speaking and listening environment that affects successful communicative exchanges. Now, we need to seek potential solutions to either alleviate or solve this communicative dilemma.

### The Effects of Wearing Transparent Masks

In the study, our transparent mask (i.e., ClearMask) had a transparent, wide, plastic barrier that enabled listeners to observe a speaker's facial expressions and mouth and lip movements in face-to-face communication (see Figure 1). Our results showed significant interactions between the presentation modes and the types of face masks worn. As expected, the no mask condition ranked the highest for speech intelligibility in both AO and AV presentations. Disposable face masks ranked second for speech intelligibility in the AO presentation and third for the AV presentation. In the AO condition alone (absence of visual information), attenuation of acoustic signals could have contributed to the decrease of speech intelligibility for transparent masks. On the contrary, transparent masks ranked second for speech intelligibility in AV presentation and third for AO presentation. These results emphasize the importance of visual information in efficient communication; thus, we see there are benefits to using transparent masks in environments that allow individuals to have visual access during their communicative exchanges.

Our result is consistent with Atcherson et al. (2017) who examined the effects of transparent masks on speech intelligibility in individuals with and without hearing loss. Both listeners with and without hearing loss benefitted from visual input from a transparent mask based on the comparison between auditory only and audiovisual presentations. Despite significant acoustic attenuation (Corey et al., 2020), the transparent mask enhanced speech intelligibility by providing visual information in AV conditions. We found that there was not a significant difference between the transparent mask vs. no mask condition when the talker in this study used clear speech in the presence of SSN in the AV presentations. This lack of difference is notable because it suggests that when a talker uses clear speech mixed with energetic maskers (i.e., white noise) while wearing a transparent mask, his/her speech is approximately as intelligible as a talker in the same setting without a face mask. This finding indicates that talkers may be able to alleviate communicative challenges affecting speech intelligibility by wearing a transparent mask and using clear speech.

In contrast, the disposable face mask did not improve speech intelligibility in spite of listeners' access to partial facial expressions including eye and eyebrow movements in the presence of SSN in the AV presentations (see Figure 4). Disposable face masks cover the mouth and lip gestures that are critical aspects of verbal communication. Visual access to extraoral facial movements alone was not enough to improve speech intelligibility in the presence of SSN. However, in the presence of 4-T speech intelligibility improved when listeners could see the talker's eyebrow movements even though the talker's lip and jaw movements were covered by a disposable face mask (see Figure 4). Informational maskers (i.e., 4-T) increase the difficulty of segregating target stimuli from background noise; however, to combat this barrier, upper facial gestures by the talker's animated eye and eyebrow movement might facilitate the listeners' comprehension by using the cues to determine the beginning of each sentence. Upper facial expression such as eyebrow movement is associated with prosodic information and people utilize talker's upper facial gestures as a cue to determine an intonation pattern or stressed word of an utterance (Lansing and McConkie, 1999; House et al., 2001; Swerts and Krahmer, 2008). While the current study was not experimentally designed to examine particular facial cues on speech intelligibility, the talker's spontaneous eyebrow movements may have helped listeners detect keywords by providing prosodic cues.

Our results support that visual information including lip gestures, jaw movements, eye, and eyebrow movement is a crucial component for comprehensibility (Sumby and Pollack, 1954; Erber, 1975; Buchan et al., 2008; Van Engen et al., 2014). These findings can provide useful and applicable evidence and resources for choosing a transparent mask for communicative exchanges especially for individuals (e.g., individuals with hearing loss) who need to see talker's articulatory movements and facial expressions to understand spoken words in noisy environments such as a busy emergency room. If a healthcare worker needs to communicate face-to-face with a patient's loved one or co-worker in the midst of a noisy environment, she would be better understood by her communication partner when wearing a transparent mask because her listener could take advantage of visual speech cues and facial expressions.

### The Effects of Clear Speech

Clear speech benefits on speech intelligibility have been well-documented and evaluated by a variety of procedures prior to the COVID-19 pandemic (Bradlow and Bent, 2002; Bradlow et al., 2003; Ferguson, 2012; Smiljanic and Sladen, 2013; Ferguson and Quené, 2014; and Van Engen et al., 2014; Smiljanic and Gilbert, 2017b; Rodman et al., 2020). Findings presented in previous literature motivated further evaluation of clear speech benefits but within simulated environments such as the ones we are currently facing in this problematic communication climate. Our results revealed that clear speech results in higher speech intelligibility than conversational speech in all types of mask conditions in both background noises and presentation modes (see Figure 5). The only exception was found in the contrast of clear vs. conversational speech produced with a transparent mask in 4-T presented in the AO condition most likely due to the “floor effect.” These findings are mostly in agreement with Keerstock et al. (2020) who found that the use of clear speech improved intelligibility produced with no mask and disposable face masks in the presence of two maskers. The improvement was also found in both native and non-native English talkers compared to conversational speech. Our listeners yielded similar keyword identification performances in the following two conditions: no mask with CL in SSN in the AO mode and disposable face mask with CL in SSN in the AO mode (see Figure 3). According to this result, with communication via auditory mode, minimal acoustic attenuation (Corey et al., 2020) is compensated with the use of clear speech. Our acoustic analysis of the speaker's sentences also showed that clear speech enhanced acoustic signals. Acoustic signals became more audible when the speaker intentionally used clear speech by slowing her speech rate and magnifying her pitch variations. Clear speech also reduced the loss of acoustic energy in higher frequencies between 3 and 10 kHz compared to conversational speech (see Figure 2). The preservation of energy in higher frequencies may lead to the comparable speech intelligibility results that were observed between the no mask and disposable face mask condition (Monson et al., 2014). The comparability between the no mask and disposable face mask condition indicates that clear speech could be a solution to overcome poor intelligibility caused by a lack of visual cues. Based on the findings, clear speech may facilitate the understanding of talkers who wear face masks when talking through the phone. For example, in the environments where people are required to wear face masks at work and need to talk over the phone, the use of clear speech may be helpful for listeners' comprehension. For the AO condition with the presence of 4-T, the only contrast that did not show clear speech benefits was CO and CL produced via the transparent mask. In this condition, a majority of listeners performed very poorly when identifying keywords in the sentences (The average and range values of keyword identification across the listeners for CO and CL produced with TM in 4-T and AO are as follows. CO: 9%, 0–30%, CL: 7%, 0–20%). These significantly lower scores are indicative of a “floor effect.” At this level of performance, it is difficult to differentiate a precise effect of clear speech.

Furthermore, there were no significant differences in speech intelligibility in the AV presentations between no mask and the transparent mask conditions with CL in SSN (see Figure 3). This result indicates that the use of clear speech can overcome a substantial amount of degraded acoustic signals imparted by transparent masks as long as visual information remains preserved for the listener/communication partner. Despite the greater degradation of the acoustic signal with a transparent mask and informational masker, listeners' performance substantially improved for clear speech when listeners could see the visual information in the AV presentation (see Figures 4, 5). Van Engen et al. (2014) revealed a significantly larger audiovisual effect observed in clear speech compared to conversational speech. In conditions when target speech is masked by competing speech and degraded acoustic signals, clear speech and facial expression cues could aid listeners in focusing on target speech and conquering reduced “glimpses,” spectro-temporal regions in which the target signal is least affected by background noise (Cooke, 2006). This finding also supports the assertion that clear speech aids listeners in their efforts to utilize and take greater advantage of visual cues. The clear speech effect is enhanced when listeners can see visual speech cues. During face-to-face conversations in noisy environments, we can take advantage of using clear speech and transparent face masks to enhance speech communication.

### Limitations and Future Directions

In face-to-face conversation, various factors regarding speakers and listeners variability affect audiovisual speech intelligibility (e.g., Hazan et al., 2010). The current study included one native, female, English speaker who produced all target sentences, neutralizing potential effects of talker diversity. The speaker was one of the authors of this paper. However, to minimize her knowledge of the study's purpose, the speaker was asked to record the target sentences using conversational and clear speech while remaining unaware of the research hypothesis initially drafted by the first author. The speaker recorded the experimental materials before being further informed of the purposes and aims of this study. However, while she was naïve to the specific hypotheses, she was aware of the general goals of research in the department, and her unconscious biases may have affected her speech productions. Ideally, future studies would recruit speakers who are not associated with the research study and are purely naïve. Our study lacked a diversity of listeners (i.e., majority were TTUHSC students). Previous research has shown that talker's race, ethnicity, and accented speech can affect audiovisual speech intelligibility (Hazan et al., 2010; Yi et al., 2013; Babel and Russell, 2015). Our listeners may have identified the speaker's productions at higher accuracy due to sharing similar cultural backgrounds/experiences and southern dialect. Listeners may have had more difficulty understanding non-native speakers who wear face masks due to different accents and listeners' cultural biases. Our study included listeners who were primarily adults from Texas. A limited diversity in listeners' cultural backgrounds may have affected the study results. Additionally, all listeners were in their early twenties except one (one older listener's score was comparable to the other participants). A preprint study revealed an older group of listeners showed lower audiovisual speech intelligibility scores compared to a group of young adults, and the older listeners subjectively rated that they required more effort to listen to talkers who wore masks compared to young adults (Brown et al., 2021). These findings suggest that age may affect speech intelligibility scores. In addition, individuals may have negative attitudes toward wearing face masks (Taylor and Asmundson, 2021). Particular attitudes (negative or positive) could affect how one understands a speaker who wears a mask. Listeners' experiences in multicultural environments and their attitudes toward wearing face masks may lead to different speech intelligibility results. Future studies should attempt to gather a larger pool of speakers and listeners with varying ages, home languages, and cultural backgrounds. Additionally, future studies could use questionnaires to determine listeners' attitudes, cultural experiences, and biases. Researchers could assess the effect of listener differences on audiovisual speech intelligibility when a mask is worn by a diverse group of speakers.

Additionally, the current study only included neurotypical listeners without hearing loss. Individuals with disabilities, hearing loss, and second language learners were excluded from the study. The CDC recommends using a transparent mask when communicating with special populations such as those mentioned above. Future studies should consider recruiting individuals of specific populations who need to see facial expressions, especially mouth and lip movements, to examine the impact of face masks on communication for these groups.

Lastly, our study's method design does not necessarily replicate a natural face-to-face interaction due to our controlled experimental conditions. Experiments were conducted with recordings in a sound booth with limited distractions (i.e., no pictures or individuals moving in the background) and we zoomed in on the speaker's face for the listeners to clearly observe the speaker. These conditions do not necessarily represent all realistic face-to-face communicative exchanges. It is important to consider that realistic face-to-face interactions incorporate a variety of factors that help speakers transmit their messages effectively. Talkers typically include gestures and other non-verbal expressions (e.g., hand gestures) to transmit their messages effectively (e.g., beat gestures, McNeill, 1996). McNeill (1996) found that hand gestures facilitated non-native language speech perception and enhanced social evaluations when the message was conveyed with hand gestures (Billot-Vasquez et al., 2020). However, our research design did not evaluate or incorporate the inclusion of hand gestures or any other non-verbal expression (i.e., body language observed below the speaker's chest) on speech intelligibility. Our audiovisual stimuli did not capture the speaker's hand gestures and body movements when producing target sentences. Additionally, we examined speech intelligibility in background noise with a −5 SNR. In face-to-face conversations, people encounter a variety of background noises with fluctuating noise levels. We encounter communication environments with drastic SNR levels (i.e., noisy settings) and quiet settings. Future studies should include different SNR levels to reflect a diversity of common communicative situations. Lastly, we only examined two types of masks; a transparent face mask and a disposable face mask. People use various types of face masks and each face mask attenuates acoustic signals differently (Corey et al., 2020). Our results may not be transmittable to other face masks that are not classified as the transparent masks or disposable face masks used in this study. Various face masks may affect audiovisual speech intelligibility differently. Future studies should consider exploring how various types of face masks impact speech intelligibility. Despite these limitations, the results of the current study can provide guidelines to improve communication while wearing face masks during and after the COVID-19 pandemic.

## Conclusion

Effects of wearing face masks on speech intelligibility in the presence of noise and the benefits of transparent masks and clear speech on speech intelligibility were evaluated. Results indicate that the combination of face mask type and the presence of background noise negatively impacts speech intelligibility in normal listeners. Audiovisual cues available with transparent masks facilitate the ability to decipher and comprehend speech in the presence of both types of background noises. The inclusion of visual information offered by the transparent mask significantly helped these listeners understand the target sentences. Lastly, our findings disclosed the benefits of clear speech on speech intelligibility in all mask conditions. Using clear speech could alleviate challenging communication situations including lack of visual cues and reduced acoustic signals. Adding this type of information as well as ways to accomplish clear speech might be helpful for speakers and listeners in enhancing intelligibility in mask wearing conditions.

## Data Availability Statement

The datasets presented in this study can be found in online repositories. The names of the repository/repositories and accession number(s) can be found below: Face mask study database, https://osf.io/hg82c/.

## Ethics Statement

The studies involving human participants were reviewed and approved by Texas Tech University Health Sciences Center Lubbock/Odessa Institutional Review Board. The patients/participants provided their written informed consent to participate in this study. Written informed consent was obtained from the individual(s) for the publication of any potentially identifiable images or data included in this article.

## Author Contributions

HY conceptualized, designed the study, and supervised all stages of the project. HY and AP carried out the behavioral experiments and drafted the full paper. WS formulated stimuli, provided guidance on the statistical approach arena, and reviewed all analyses. All authors discussed the results and implications and contributed to the manuscript.

## Conflict of Interest

The authors declare that the research was conducted in the absence of any commercial or financial relationships that could be construed as a potential conflict of interest.
