# Supplementary material for: Effects of Wearing Face Masks While Using Different Speaking Styles in Noise on Speech Intelligibility During the COVID-19 Pandemic
Source: Front Psychol. 2021 Jun 28;12:682677. doi: 10.3389/fpsyg.2021.682677 (PMC8292133; doi:10.3389/fpsyg.2021.682677)
Supplement: Supplementary file 1 [file Data_Sheet_1.docx]

| No | Sentence |
| --- | --- |
| 1 | These BROWN MUSHROOMS TASTE AMAZING. |
| 2 | The WEAK PLANT is BARELY ALIVE. |
| 3 | Our CAT HATES TAKING a BATH. |
| 4 | The BIG ROOM FELT EMPTY. |
| 5 | His GRANDMA and GRANDPA HELPED the KIDS. |
| 6 | The SICK PERSON FEELS BETTER. |
| 7 | The COOL NIGHT was COMFORTABLE and RELAXING. |
| 8 | The SECRETARY LEARNED SPANISH EASILY. |
| 9 | The BEDROOM RUG had a LARGE STAIN. |
| 10 | My YELLOW SHOES CAME in a BAG. |
| 11 | The RATS RAN through the DARK STREETS. |
| 12 | The HONEST MOTHER is LOVING and NICE. |
| 13 | The BLUEBERRY PIE BAKED in the OVEN. |
| 14 | The HAPPY CHILDREN LAUGH at the STORY. |
| 15 | The FATHER and DAUGHTER SAW the MOVIE. |
| 16 | Her FAVORITE PANTS WERE RUINED. |
| 17 | She WASHED and DRIED her CURLY HAIR. |
| 18 | A GOOD FRIEND TELLS the TRUTH. |
| 19 | The FOREIGN LADY DREAMED of her HOME. |
| 20 | The STARVING DOG SMELLED the FOOD. |
| 21 | Our COUSIN STARTS SCHOOL TOMORROW. |
| 22 | The MEAN TEACHER is NEVER NICE. |
| 23 | The HOT SUN WARMED the POOL. |
| 24 | The MAN LOST his HOUSE KEY. |
| 25 | The MARKET was CROWDED and TOO FAR. |
| 26 | The RAIN LASTED for MANY WEEKS. |
| 27 | Her LOUD COUGH SOUNDED HORRIBLE. |
| 28 | His SPEECH was BORING and TOO LONG. |
| 29 | The FIVE STUDENTS were LATE for CLASS. |
| 30 | The PRIVATE UNIVERSITY is NOT CHEAP. |
| 31 | The STRONG ARMY WON the BATTLE. |
| 32 | That BOOK COST TEN DOLLARS. |
| 33 | The CHEF MADE FRESH NOODLES. |
| 34 | The CLEAN BEACHES have CLEAR WATER. |
| 35 | The YOUNGEST SISTER WATCHES TV. |
| 36 | The CHEAP DRINKS ATTRACT CUSTOMERS. |
| 37 | The ADULTS LEARNED to DANCE in SCHOOL. |
| 38 | The STUDENT STUDIES in the QUIET ROOM. |
| 39 | The STRESSFUL WEEK ENDED at the BAR. |
| 40 | The GIRL LOVES SWEET CANDY. |
| 41 | The CHEF COOKS PASTA EVERY day. |
| 42 | His FIRST GIRLFRIEND was ATTRACTIVE and SMART. |
| 43 | The KING and QUEEN PLANNED a PARTY. |
| 44 | The GOAT EATS DRY LEAVES. |
| 45 | The TALENTED ARTIST DREW a PICTURE. |
| 46 | The SHY GUEST SPEAKS QUIETLY. |
| 47 | The CHICKEN SANDWICH CAME with SALAD. |
| 48 | The VEGETABLES GREW in the GREEN GARDEN. |
| 49 | The GROUP HEARD SLOW MUSIC. |
| 50 | The THIRSTY KID DRINKS JUICE. |
| 51 | The NEWSPAPER COMES EVERY WEEKEND. |
| 52 | The CHICKEN SOUP was a TASTY MEAL. |
| 53 | The AIRPLANE FLEW in the BLUE SKY. |
| 54 | The BROWN BEARS EAT FRUIT. |
| 55 | The TWO WAITERS SERVED BREAKFAST. |
| 56 | The SPICY CARROTS were her FAVORITE DISH. |
| 57 | The LIGHTNING ENDED the SOCCER GAME. |
| 58 | My DOCTOR WORKS in that BUSY HOSPITAL. |
| 59 | The TRAVELERS VISITED the ART MUSEUM. |
| 60 | The COMPANY BUYS FOREIGN CARS. |
| 61 | The FAMILY CELEBRATED their FAVORITE HOLIDAY. |
| 62 | The RICH DENTIST BOUGHT new TOOLS. |
| 63 | The RELIGIOUS COUPLE BELIEVES in GOD. |
| 64 | The TWIN SISTERS WATCHED a MOVIE. |
| 65 | The LAST YEAR was CALM and PEACEFUL. |
| 66 | The LIGHT BLUE JEANS are not EXPENSIVE. |
| 67 | The RICE and BEANS LOOKED DRY. |
| 68 | The BIRD FLEW OVER the SEA. |
| 69 | The JAZZ SINGER SOUNDED GREAT. |
| 70 | The BIRD FOUND a JUICY WORM. |
| 71 | The BUSY FARMER GROWS POTATOES. |
| 72 | The SMALL BOY SEEMED SAD. |
| 73 | The PROUD FANS CHEERED for their TEAM. |
| 74 | The STEAMED CHICKEN TASTED STRANGE. |
| 75 | The SINGER and DANCER JOINED the ACTOR. |
| 76 | The CUSTOMERS HATE BLACK TEA. |
| 77 | Those CUTE ANIMALS CHEWED the PLANTS. |
| 78 | A BASEBALL is HARD and PERFECTLY ROUND. |
| 79 | The MATH TEST was EASY to FINISH. |
| 80 | The MOUSE FOUND TASTY CHEESE. |
| 81 | The KIDS SCREAMED LOUDLY in the PARK. |
| 82 | The PLANTS and TREES LOOK BEAUTIFUL. |
| 83 | A SUMMER VACATION is ALWAYS RELAXING. |
| 84 | The CLASS LEARNED about EARTH SCIENCE. |
| 85 | They ATE the ENTIRE CABBAGE QUICKLY. |
| 86 | The RED GRAPES are BIG and TASTY. |
| 87 | The SUN SHINED BRIGHTLY in JUNE |
| 88 | The CHURCH GROUP INSPIRED the COMMUNITY. |
| 89 | The MEETING STARTS in TWENTY MINUTES. |
| 90 | The STARS LIT the NIGHT SKY. |
| 91 | The TEENAGER LIFTED a HEAVY BOX. |
| 92 | The FISH SWAM SLOWLY in the LAKE. |
| 93 | The BUSINESS CREATED MANY JOBS. |
| 94 | The MEAN PEOPLE BROKE the RULES. |
| 95 | The FUNNY MOVIE will END SOON. |
| 96 | The WORKER HURT his LEFT HAND. |
| 97 | The SOFT MUSIC PLEASED EVERYONE. |
| 98 | The PLAYERS FORGOT to bring LUNCH AGAIN. |
| 99 | The HUGE SUPERMARKET OPENS TOMORROW. |
| 100 | The ARTIST VISITED MANY MUSEUMS. |
| 101 | The FLAGS FLY HIGH and PROUD. |
| 102 | The MACHINE MADE a TERRIBLE NOISE. |
| 103 | Our BAND PRACTICES in my SMALL GARAGE. |
| 104 | Her UNCLE WAITS QUIETLY for the ANSWER. |
| 105 | The MAP SHOWS the CITY ROADS. |
| 106 | The STRONG WIND COOLED the AIR. |
| 107 | The POPULAR CLUB is OFTEN FULL. |
| 108 | The PAINTER USED SOFT BRUSHES. |
| 109 | My BROTHER SLEEPS until LATE MORNING. |
| 110 | The RABBIT and MOUSE EXPLORED the FIELD. |
| 111 | The HUNGRY TEENAGERS EAT SNACKS. |
| 112 | The ANGRY BEAR SCARED the CAMPERS. |
| 113 | The SAD PETS NEED LOVE. |
| 114 | He LOST his WHITE HAT TODAY. |
| 115 | The THREE COUSINS did their MATH HOMEWORK. |
| 116 | The CROWD WATCHED the TALENTED PERFORMER. |
| 117 | My GRANDPARENTS TOOK PICTURES on VACATION. |
| 118 | The WHITE HORSE LIVES on a FARM. |
| 119 | That PRETTY GIRL WON a PRIZE. |
| 120 | The GARDENER GREW COLORFUL PEPPERS. |
